# Aquifer systems extending far offshore on the U.S. Atlantic margin

**DOI:** 10.1038/s41598-019-44611-7

**Published:** 2019-06-18

**Authors:** Chloe Gustafson, Kerry Key, Rob L. Evans

**Affiliations:** 1 Lamont-Doherty Earth Observatory, Columbia University, Palisades, New York USA; 2 Department of Geology and Geophysics, Woods Hole Oceanographic Institution, Woods Hole, Massachusetts USA

**Keywords:** Hydrology, Geology, Geophysics, Hydrogeology

## Abstract

Low-salinity submarine groundwater contained within continental shelves is a global phenomenon. Mechanisms for emplacing offshore groundwater include glacial processes that drove water into exposed continental shelves during sea-level low stands and active connections to onshore hydrologic systems. While low-salinity groundwater is thought to be abundant, its distribution and volume worldwide is poorly understood due to the limited number of observations. Here we image laterally continuous aquifers extending 90 km offshore New Jersey and Martha’s Vineyard, Massachusetts, on the U.S. Atlantic margin using new shallow water electromagnetic geophysical methods. Our data provide more continuous constraints on offshore groundwater than previous models and present evidence for a connection between the modern onshore hydrologic system and offshore aquifers. We identify clinoforms as a previously unknown structural control on the lateral extent of low-salinity groundwater and potentially a control on where low-salinity water rises into the seafloor. Our data suggest a continuous submarine aquifer system spans at least 350 km of the U.S. Atlantic coast and contains about 2800 km^3^ of low-salinity groundwater. Our findings can be used to improve models of past glacial, eustatic, tectonic, and geomorphic processes on continental shelves and provide insight into shelf geochemistry, biogeochemical cycles, and the deep biosphere.

## Introduction

Offshore groundwater plays an important role in continental shelf systems, with implications for past and present environments. Submarine groundwater discharge, the flow of water from continents to the ocean via onshore outcrops and submarine channels, affects the biologic and chemical processes of the ocean, which in turn may regulate nutrient fluxes important for global carbon cycling^1^. Sequestered submarine groundwater emplaced in continental shelves during past sea-level low stands may provide information regarding past ocean salinity changes^2^ and may also be used to constrain paleo-hydrologic models that give insight into past glacial extent and sea-level changes^3–6^. Whether modern or ancient, low-salinity groundwater also represents a potential resource in regions where onshore freshwater resources have diminished.

There is ample evidence of low-salinity submarine groundwater contained within the world’s continental shelves, with direct or indirect observations occurring on all continents^7^. Several of these observations have taken place on the U.S. Atlantic coast. During the 1970’s, the AMCOR drilling project revealed low-salinity groundwater up to 120 km offshore New Jersey. This dataset resulted in the development of a model of offshore low-salinity groundwater characterized by a wedge-shaped feature, gradually increasing in salinity seaward and with depth^8^. A more focused drilling survey completed by IODP Expedition 313 in 2010 showed more complex groundwater geometry offshore New Jersey^9^, proving the earlier AMCOR-based model too simple. Further northeast, low salinities seen in well data from onshore Martha’s Vineyard^10^ and Nantucket^11^ have also suggested low-salinity submarine groundwater is present, although there has been no offshore drilling to confirm this hypothesis. While borehole data provide direct samples, they are only spatial point measurements and do not provide a laterally continuous characterization of the subsurface necessary for understanding offshore groundwater distributions.

Paleo-hydrologic modeling combines stratigraphy with past sea-level and ice-sheet history to better understand offshore groundwater. Modeling studies have attempted to characterize the spatial distribution of low-salinity groundwater offshore the U.S. Atlantic coast^3–6^, especially in regions with no direct offshore observations, such as Martha’s Vineyard. These models all provide different predictions of groundwater characterization due to variations in model forcings and hydrological parameters, resulting in significant uncertainty in the nature of the groundwater that may be present within the U.S. Atlantic margin.

**Figure 1:**
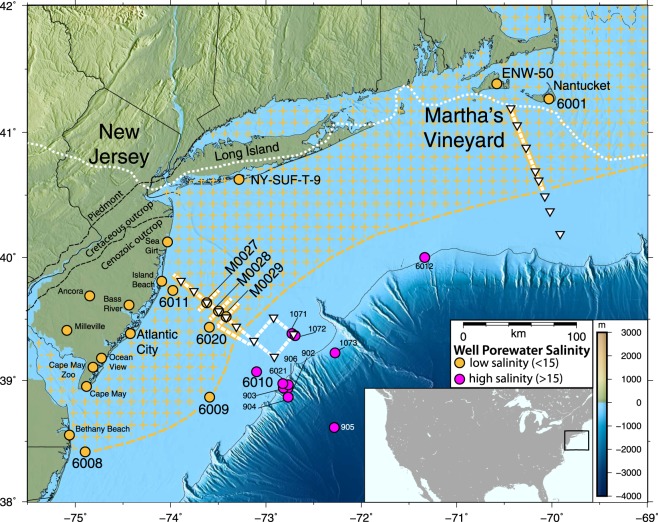
Electromagnetic survey of groundwater on the U.S. Atlantic continental shelf. Surface-towed CSEM profiles (white dashed lines) and seafloor MT stations (white triangles) were collected during surveys offshore New Jersey and Martha’s Vineyard. Yellow solid lines show the portions of the survey profiles where the EM data support low-salinity pore water. Circles denoting onshore and offshore wells^8–11,42–45^ are colored yellow where low pore fluid salinities (<15) were observed offshore or where aquifers have been identified via geophysical data onshore. Magenta circles denote where only high salinity (>15) pore waters have been encountered. Wells featured in the text are labeled with a larger font size. Yellow crosses and the yellow dashed line show our inferred spatial extent of the low-salinity aquifer system. The white dotted line denotes the terminal moraine of the Laurentide ice-sheet at the Last Glacial Maximum^30^. This map was created using GMT-5.4.3 (http://gmt.soest.hawaii.edu/projects/gmt/wiki/Download). The topography source data for this figure is the Global Multi-Resolution Topography Data (https://www.gmrt.org).

Marine electromagnetic (EM) methods are geophysical methods capable of providing continuous measurements required for characterizing offshore groundwater systems. EM methods are sensitive to bulk electrical resistivity, which is primarily controlled by porosity and pore fluid salinity^12^ in offshore sediments. Many authors have called for the utilization of EM methods to better understand offshore groundwater systems^5–7^ and active-source EM studies over small spatial scales have successfully identified offshore groundwater^13,14^. Our study implemented technology originally produced for hydrocarbon exploration^15^ in order to establish the first large-scale marine EM survey of low-salinity groundwater. We used a recently developed surface-towed controlled source EM (CSEM) system^16^ in combination with the passive-source magnetotelluric (MT) method to map low-salinity groundwater systems in two locations on the US Atlantic coast: offshore Martha’s Vineyard and New Jersey (Fig. 1). CSEM and MT methods are preferentially sensitive to resistors and conductors, respectively (Methods), so we use both data sets to obtain a more robust resistivity model.

Offshore New Jersey, our profiles were designed to better understand the spatial extent of low salinities already measured by the nearby AMCOR wells (6011, 6020; Fig. 1) and the co-located IODP Exp. 313 wells (M0027, M0028, M0029; Fig. 1), while offshore Martha’s Vineyard we collected a profile to test whether similar low-salinity anomalies are present, as suggested by nearby onshore wells (6001 and ENW-50 (Fig. 1)). At both locations we collected continuous surface-towed CSEM data along 130 km long profiles extending from the shoreline to the shelf edge. Eight MT receivers were deployed 10–20 km apart along each main transmission profile to constrain the broad-scale structure. Offshore New Jersey, two additional receivers were deployed 20 km northeast of the seaward edge of the main transmission profile and we also collected CSEM data along seven additional intersecting profiles (Fig. 1).

We carried out independent and joint inversions of the MT and CSEM data using a standard regularized 2-D inversion code that yielded resistivity models for each profile (Methods and ref.^17^). We use our resistivity models to infer the distributions of low- and high- salinity water in the subsurface. Here we focus on the top 800 m of the models where the resolution is highest and where we expect low-salinity water. For the purpose of discussion within this text, we define low- and high- salinity water as having salinity less than and greater than 15, respectively, where salinity is a unitless quantity as defined by the Practical Salinity Scale^18^.

**Figure 2:**
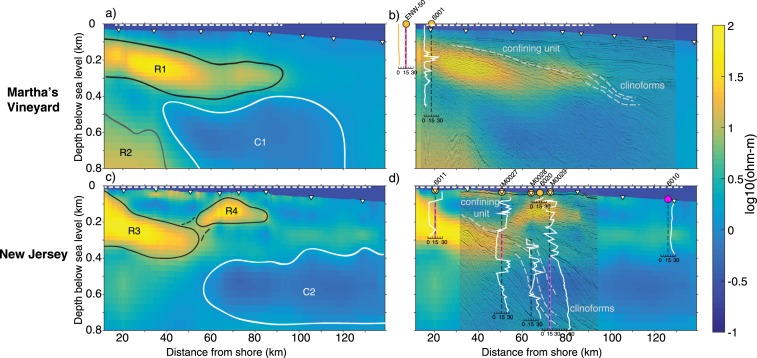
Resistivity models obtained from jointly inverting surface-towed CSEM and seafloor MT data for the shore-to-shelf profiles off of Martha’s Vineyard and New Jersey with seismic and well log data. **a-d**, Shaded colors show high resistivity as yellow hues and low resistivity as blue hues. White triangles show seafloor MT receiver locations and dashed white lines show the extent of the surface-towed CSEM data used in the inversions. The left column shows the resistivity models for (**a**) Martha’s Vineyard and (**c**) New Jersey overlain with black lines showing resistive zones interpreted as low-salinity aquifers and white lines showing the deeper conductive zones interpreted as saline or briny water. The deeper Jurassic basement seen in seismic reflection data collected off Martha’s Vineyard is shown by a dark grey line. The right column displays co-located seismic reflection images on top of the (**b**) Martha’s Vineyard and (**d**) New Jersey resistivity models as well as pore water salinity data^8–11^. Salinity data are plotted as white lines on linear scales. Onshore well ENW-50 salinity data are plotted in yellow. Black dashed lines represent a salinity value of 15. Solid pink and purple lines on salinity plots are the depths and corresponding salinity values used in Fig. 3. Seismically-imaged confining units and clinoform structures that influence groundwater distribution patterns are shown as labeled light grey dashed lines.

Offshore Martha’s Vineyard, our model (Fig. 2a,b) contains two resistive zones and one conductive zone. The resistive and conductive zones range from 10–70 ohm-m and 0.6–1 ohm-m, respectively. Resistive zone R1, located within the top 400 m, gently dips seaward out to 90 km offshore and is approximately 200 m in thickness. R1 is partially underlain by conductive zone C1, which spans from 40–120 km offshore and reaches depths of at least 800 m. The deeper near-shore resistive zone, R2, is consistent with a Jurassic basement rock interpreted in a co-located seismic image^6^.

Off New Jersey, our resistivity model (Fig. 2c,d) reveals two resistive zones within the upper 400 m that are underlain by a conductive zone. The resistive and conductive zones range from 10–110 ohm-m and 0.4–1 ohm-m, respectively. The near-shore resistive zone, R3, shallowly dips downward from the shore out to 60 km offshore, reaching a maximum depth of about 400 m. The mid-shelf resistive zone, R4, is imaged in the top 50–200 m and extends from 60 km to 90 km offshore, with no obvious dip. The underlying conductive zone C2, ranges from 450–750 m depth and spans from 60 km offshore to the end of the profile. Additional resistivity images for our shoreline parallel profiles show that the resistive and conductive zones extend in the shore-parallel directions for the entire lengths of the crossing profiles (Extended Data Fig. 8).

**Figure 3:**
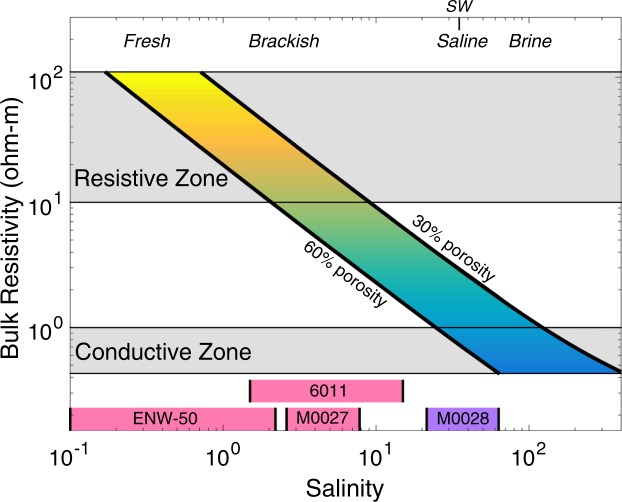
Dependence of bulk resistivity on salinity. Bulk resistivity calculated as a function of pore water salinity and porosity using Archie’s Law is shown as thick black lines. Salinity is shown using the unitless Practical Salinity Scale 1978^18^. Porosity values ranging from 30–60% are representative of the range encountered in wells M0027–M0029^9,20^. Shaded colors denote resistivity with the same color scale as Fig. 2. Grey horizontal bars show resistive and conductive zones identified in Fig. 2. The combination of observed resistivity and porosity indicates that there is low-salinity pore water in the resistive zone and saline or briny pore water in the conductive zone. Water salinity classifications are shown on the top of the horizontal axis with seawater (SW) specifically noted at the value 35. Select low- and high- salinity ranges from co-located wells are plotted as pink and purple bars, respectively. The salinity ranges are limited to depths where the corresponding wells intersect the resistive and conductive zones, shown in Fig. 2b,d. These ranges show good agreement with our imaged resistivity for both the resistive and conductive zones.

We infer pore water salinity by combining measured porosities with our resistivity models and employing standard relationships between pore fluid resistivity, salinity and porosity^12,18^. These relationships demonstrate that areas of high and low resistivity should correspond to areas of low- and high- salinity pore water, respectively (Methods). Borehole sediment analyses from IODP Exp. 313 shows that low-salinity waters exist in sediments with average porosities ranging from 30–60%^19^, so we use these values in our conversion from resistivity to salinity. The resistivity values of R1, R3, and R4 suggest pore water salinity values range from about 0.2–9 (Fig. 3), in good agreement with salinity ranges observed in intersecting wells^9,20,21^. The deeper conductive zones in both profiles require salinities that exceed that of seawater (Fig. 3). Our imaged resistivity variations could also be explained by porosity variations alone; however, if the pore fluid has seawater salinity, resistive zones R1, R3 and R4 would have only 2–10% porosity, which is significantly lower than the borehole observations. Instead, our preferred interpretation is that resistive zones R1, R3 and R4 represent low-salinity groundwater and conductive zones represent saline or briny groundwater in the subsurface.

We interpret our resistive zones as submarine aquifers given observed or predicted permeabilities for both New Jersey and Martha’s Vineyard. Previous work combining IODP Exp. 313 sediment and seismic profile analyses offshore New Jersey predicts permeabilities ranging from 10–1000 md for offshore New Jersey^19^. Permeability measurements from onshore Nantucket (6001) range from 10–780 md^11^. Although permeabilities vary, all permeability values are high enough to allow the flow of water through sediments^19,22^, justifying our use of the term “aquifer” to describe our resistive zones.

Direct pore water salinity measurements offshore New Jersey correspond well with our resistivity model (Fig. 2d). The upper and lower boundaries of R3 show excellent agreement with low-salinity measurements from wells 6011^8^ and M0027^9,20^,while high-salinity measurements from wells M0027, M0028, and M0029^9,20^ are consistent with the low resisivities of C2. The correlation between direct salinity measurements and resistivity is not as strong for R4, however, wells 6020^8^ and M0029^9,20^ both show a sharp increase in pore water salinity at the upper boundary of R4. Further seaward, well 6010^8^ is also compatible with our resistivity model, as there are no low-salinity measurements present where we do not image high resistivity.

No well data exists offshore Martha’s Vineyard, however, we can compare our offshore resistivity model with nearby onshore well data from Martha’s Vineyard^23^ (ENW-50) and Nantucket^21^ (6001) (Fig. 2b). Well ENW-50 shows low salinities over its entire depth range, which extends from the surface down to 228 m. This correlates well with the location of R1 and strongly suggests that R1 connects onshore. This region of low-salinity is also seen in 6001, but is expressed at greater depths (below 320 m) due to the thinning of sediments from Natucket to Martha’s Vineyard^10,24^.

Seismic reflection images^6,25^ co-rendered on our models (Fig. 2b,d) reveal consistent structural controls on the low-salinity groundwater distribution. Continuous, well-defined stratigraphic boundaries mark the upward extent of low-salinity groundwater and the onset of clinoform structures mark the seaward extent for R1 and R3. While it is known that groundwater systems are typically constrained vertically by a confining unit, the correspondence of the low-salinity groundwater seaward terminus with the onset of clinoforms for both R1 and R3 demonstrates a previously undetermined structural control on offshore groundwater distribution. Notably, both confining units mark a change in geology; the top bound of R1 is a known unconformity^26^ and the top bound of R3 marks the transition from continuous to discontinuous seismic reflectors, indicating marine and non-marine sediment deposition, respectively.

Seismic reflection images combined with our resistivity models and well data strongly suggest that both R1 and R3 connect onshore. Resistors R1 and R3 have high-resistivity values (and therefore low-salinity) at the shoreward extent of both profiles, suggesting a connection to the onshore system. Offshore New Jersey, the highest resistivity values occur at the most landward extent of our profile, which is consistent with lateral transport of freshwater from onshore recharge. The seismically imaged aquifer confining units also continue to the shoreward bounds of our images (Fig. 2b and ref.^19^), providing further evidence for an onshore connection. Previous work combining IODP Exp. 313 sediment and seismic profile analyses indicates R3 exists within low-permeability sediments that continue onshore New Jersey^19^, where there are several known aquifers^27^. Moreover, onshore analysis of pore water sediments from the Atlantic City well show low-salinity water in low-permeability seaward dipping sediments at depths corresponding to R3^28^. Additionally, stable hydrogen and oxygen isotope data^20^ from IODP Exp. 313 wells indicate the low-salinity water within R3 is a mix of modern seawater and modern terrestrial meteoric water, which requires an onshore connection. Similar analyses have not been done for R1, as there are no borehole measurements offshore Martha’s Vineyard. However, as previously mentioned, onshore boreholes ENW-50 and 6001 provide direct evidence of onshore freshwater at depths corresponding with R1, making an onshore connection highly plausible.

Resistive zone R4 does not exhibit the same structural controls seen for R1 and R3. Above R3’s seismically inferred confining unit and clinoforms, R4 exists within less-continuous seismic reflectors, indicating the sediments hosting this groundwater were deposited in a non-marine environment, whereas the continuous seismic reflections in R3 indicate marine deposition. Also, unlike R3, the low-salinity water imaged by R4 exists primarily in units with predicted high permeability^19^. The difference in permeability and depositional environment of the host lithologies indicates the low-salinity water imaged by R4 may have a different origin than low-salinity water in R3. Alternatively, R3 and R4 may be connected. Similar to R3, stable hydrogen and oxygen isotope data^20^ from IODP Exp. 313 wells indicate the low-salinity water within R4 are a mix of modern seawater and modern terrestrial meteoric water. This supports the idea that R3 and R4 are connected, or suggests the low-salinity water imaged in R4 originates from a different onshore source, potentially connecting with known Long Island aquifers^27^. A connection between R3 and R4 could indicate that there is a relationship between clinoforms and upwelling groundwater, as R4 occurs above the seismically imaged clinoforms and we image high-resistivity values between R3 and R4. Low-salinity water may rise from R3 to R4 via unresolved faults, as low-salinity water is gravitationally buoyant compared to denser saline water.

For both surveys, the conductive zones appear to have no corresponding relationship with seismically imaged stratigraphic boundaries. However, underlying faults seismically imaged offshore New Jersey allow for the migration of buried salt deposits^29^, indicating the conductive zones in our results arise from groundwater interacting with these deposits.

Our results demonstrate a massive offshore aquifer system, extending 90 km offshore, spans the New Jersey coastline up to Martha’s Vineyard, and likely spans beyond the bounds of our survey. To the southwest of our New Jersey profile, wells 6008 and 6009 (Fig. 1) have identified groundwater with salinities less than fifteen^8^, establishing a significant southward extension of the low-salinity groundwater. Between our two surveys, observed onshore aquifers exist within seaward dipping sediments on Long Island that extend offshore. Previous hydrologic models show the extent of low-salinity groundwater terminates near the shoreline^27^; however, we assert that some aquifer water must extend several 10’s of kilometers offshore (Fig. 1) given the extent of the Laurentide ice-sheet during the Last Glacial Maximum^30^ (LGM) (Fig. 1) and its relationship to low-salinity groundwater that we have observed offshore Martha’s Vineyard. Subglacial runoff beneath and in front of the ice-sheet during the LGM is thought to have deposited freshwater around Martha’s Vineyard, as suggested by observed and modeled overpressures^3–6,21,31^, and likely emplaced low-salinity groundwater from offshore Long Island to the northeast of our Martha’s Vineyard survey. The yellow cross pattern in Fig. 1 shows our inferred low-salinity aquifer extent on the U.S. Atlantic margin.

**Figure 4:**
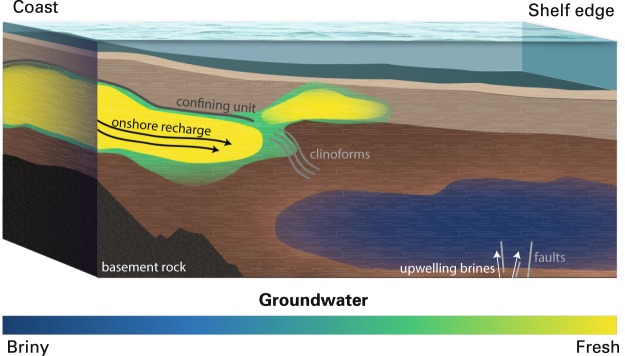
Conceptual model of offshore groundwater. Offshore low-salinity aquifers are fed by the adjacent onshore hydrologic system and are vertically constrained by a confining unit, until clinoform structures are present. Low-salinity water is also present above the clinoforms and may originate from the larger low-salinity aquifer. High-salinity groundwater exists deeper and further seaward and is caused by groundwater interaction with underlying salt deposits. Arrows denote groundwater flow paths.

The modeled size, continuity, and onshore connectedness of the low-salinity groundwater advance ideas of the offshore groundwater systems on the U.S. Atlantic coast. Previously, borehole data combined with seismic imaging were used to infer a system of multiple, localized water reservoirs within the mid-shelf offshore New Jersey^19^, whereas our models reveal a more regional view of widespread water distribution across the shelf. Offshore Martha’s Vineyard there was greater uncertainty due to an absence of data sensitive to offshore groundwater prior to our study; previous offshore models relied solely on paleo-hydrologic modeling, resulting in various hypothesized systems, ranging from isolated freshwater packets^6^ to diffusive tongues of salinity contours^3–5^. Our study shows laterally continuous low-salinity systems with well-defined boundaries in both survey locations, providing the first look at the groundwater system offshore Martha’s Vineyard and redefining the New Jersey system. We present a generalized conceptual model of offshore groundwater, including structural controls, in Fig. 4.

The spatial distribution of the low-salinity groundwater shown in our study provides important constraints for understanding continental shelf processes. Our results can be used to improve paleo-hydrologic modeling, as the interactions between stratigraphy, sea-level changes, and ice-sheet dynamics are all imprinted in submarine groundwater systems. The sequestered low-salinity groundwater we image offshore Martha’s Vineyard may be used to improve estimates of past ocean salinity changes^2^ as water stored underground and in ice-sheets impacts ocean composition. Both of our profiles may also enhance understanding of sub-seafloor ecology and microbiology, which are influenced by exchanges between low-salinity groundwater and the overlying ocean^32,33^. Furthermore, onshore connectivity of the low-salinity groundwater may represent a significant, yet widely unaccounted for, nutrient exchange mechanism between the terrestrial and ocean environments important for global carbon cycling^1^.

The low-salinity groundwater we have imaged represents a significant resource with potential for future offshore groundwater production. Given the 90 km offshore extent, 350 km distance between profiles, about 200 m thickness and 45% average porosity, we estimate the aquifer system contains at least 2800 km^3^ of water with salinity less than 15. If we consider the potential northeast and southwest extensions beyond our profiles, there may be several times more groundwater underlying the northeast portion of the U.S. Atlantic continental shelf, representing a freshwater resource that rivals the largest onshore aquifers. Utilization of such groundwater would require desalinization and effects such as upconing should be considered prior to extraction.

We have shown continuous aquifers extend from onshore New Jersey and Martha’s Vineyard out to 90 km offshore. Our data show good agreement with onshore and offshore borehole data, demonstrating marine electromagnetic data are useful for mapping offshore groundwater. Correlating our resistivity images with seismic data shows aquifers connecting onshore are vertically constrained by confining units and laterally constrained by the onset of clinoforms. Our results improve the overall knowledge of groundwater offshore the U.S. Atlantic coast and may be used in hydrologic and oceanographic models of the region in order to better understand past, present, and future continental shelf dynamics.

## Methods

The marine MT method measures the horizontal components of naturally-occurring low frequency EM fields on the seafloor to produce an impedance response sensitive to electrical resistivity from the seafloor to the deep crust and mantle, depending on the frequency range. The CSEM method uses a towed horizontal electrical dipole source to broadcast EM fields to one or more receivers that measure the attenuation and phase shift of the transmitted fields to generate an Earth response sensitive to the seafloor resistivity at depths of meters to a few kilometers, depending on the source-receiver offset and frequency band.

During the ten day cruise, we collected MT stations at each survey location by deploying ten broadband seafloor EM receivers^34^ for up to 4 days. Surface-towed CSEM data were collected by towing a 336.9 m long dipole antenna behind the ship and broadcasting a 88 A current using a doubly symmetric square waveform^35^ with a 0.25 Hz fundamental frequency. This waveform has higher signal-to-noise ratios for higher frequencies compared to a square wave and other common waveforms. The dipole moment of the antenna - the product of the antenna length and its transmitted current - is 2.96 × ${10}^{4}$ Am. This large dipole moment combined with short receiver offsets ensures the CSEM signal is stronger than MT signal^34^; MT signal manifests as lower power random noise in the measured CSEM signal. Four broadband EM receivers were surface-towed behind the vessel at offsets 600, 870, 1120, and 1380 m. Each surface-towed receiver measured the inline horizontal electric field on a 2 m dipole positioned about 0.67 m below the sea surface^16^. The receiver orientations and positions were measured using recording electronic compasses and GPS logging systems. All CSEM tows off New Jersey were completed successfully. Off Martha’s Vineyard, the shortest offset towed receiver was fouled by fishing gear and stopped producing reliable data around 10 km into the profile. At about 70 km along the profile (Fig. 1), the towed array became further damaged when snagging fishing gear and a nearby buoy line and so the surface towed data collection was terminated. Despite incurred damages, we collected 70 km of high quality CSEM data.

Extended Data Figs 1–3 show the inversion models and data from Martha’s Vineyard and Extended Data Figs 4–6 show the inversion models and data from New Jersey. CSEM response functions for the four surface-towed EM receivers were estimated with a robust stacking method^35^. We used 240 s stacking windows corresponding to 250–300 m lateral distance between transmitter stack points. This yielded high quality amplitude and phase responses for each receiver as a function of position and frequency harmonic. While the transmitter waveform yielded about 8 usable frequency harmonics, here we only consider data at the two strongest low-frequency harmonics, 0.75 and 1.75 Hz, as they contain the highest sensitivity to the depth range of interest (Extended Data Figs 3 and 6). The transmitted CSEM signal is well above the noise floor (Extended Data Fig. 7). The CSEM data from both survey regions exhibit high amplitudes and low phase lags on the inner to mid-shelf sections of the profiles, which is consistent with low attenuation associated with high resistivity seafloor in these regions. Conversely, the amplitudes significantly decrease and the phase lags increase on the outer shelf sections of the profiles, indicating the seafloor is more conductive in these regions.

MT impedance tensors were obtained by processing two to four day recordings of the seafloor horizontal electric and magnetic fields at each station using a robust multivariate array processing method^36^. This resulted in generally high-quality MT responses at periods from 0.01 to 1000 s. Broadband seafloor MT data collected in deep water (around 1 km) can typically only be obtained at periods of 1 s and longer due to the attenuation of the shorter periods by the conductive seawater^37^, but here the water depths (20–100 m) were too shallow to significantly attenuate the shorter period fields at 0.01 to 1 s. Noisy responses due to ocean waves in the 2–10 s period band were omitted from the data, depending on the station. Further, data at periods longer than 10 s were omitted from the analysis here because the depth sensitivity at periods of 10 to 1000 s greatly exceeds the depth range of interest for studying the shallow aquifers. Analysis of the Martha’s Vineyard MT profile for deep crustal and mantle structure using the full data bandwidth is presented in ref.^38^.

The resulting MT responses in the 0.01–10 s band have nearly equal off-diagonal impedance components, suggesting the shallow structure is one-dimensional within the range of sensitivity of each instrument (Extended Data Figs 2 and 5). Off Martha’s Vineyard, the MT responses at nearshore stations M01–M06 have characteristically high apparent resistivities that exceed 1 ohm-m and display a positive peak at periods less than 1 s, indicating a shallow resistive feature is present beneath these stations. The further offshore stations (M07–M10) have apparent resistivities at periods below 1 s that are closer to 1 ohm-m and without the strong peak, suggesting the absence of significant resistive structures beneath these stations. Off New Jersey, similar high apparent resistivities below 1 s period are seen for stations N01–N05, suggesting shallow resistive structure is present beneath these stations, while the outer-shelf stations (N06–N08) have much lower apparent resistivities.

Two-dimensional (2-D) resistivity models were obtained by inverting surface-towed CSEM and MT data jointly and independently using MARE2DEM^17^, a freely-available goal-oriented adaptive finite-element inversion code for 2-D electromagnetic modeling and inversion. MARE2DEM uses a standard non-linear regularized inversion approach to find the smoothest model fitting the data. We manually removed clear outliers in both the MT and surface-towed CSEM data as a final processing step before inverting the data. MT data uncertainties were subject to a 10% error floor while CSEM data were subject to a 1% error floor. We modeled the 336.9 m long transmitter antenna as a finite-length bipole by integrating point dipole sources along the length of the antenna using an efficient Gauss quadrature approach. The 2 m long towed CSEM receiver dipoles and 10 m long seafloor MT receiver dipoles were modeled using point dipole approximations. The main New Jersey profile consists of a total of 6830 CSEM data and 360 MT data. The Martha’s Vineyard profile has a total of 3260 CSEM data and 336 MT data. We constructed inversion parameter grids consisting of 31,004 cells for the New Jersey profile and 33,739 cells for the Martha’s Vineyard profile. Due to the extreme width to depth ratio in the inversion models and the ~20 km wide MT station spacing, both shore-to-shelf profiles were subject to a horizontal to vertical roughness penalty ratio of 300 while the crossing profiles were subject to a ratio of 30. All resistivity inversions fit the joint data set to a root-mean-square (RMS) misfit of 1.0.

Separate and joint inversions of the CSEM and MT data sets show the relative sensitivities to the seafloor structure (Extended Data Figs 1 and 4). The MT-only inversions show subtle resistive features in the upper 400 m from the start of the profiles to about 90 km offshore; however, the resistivity only reaches about 10 ohm-m. This is not surprising as MT data are more sensitive to thin conductive layers than thin resistive layers and MT inversions are well-known to underestimate the resistivity of thin resistive layers^39^. CSEM data are much more sensitive to resistive layers and thus the CSEM-only inversions find much higher resistivities. Further, they have much better lateral resolution due to the relatively short spacing between data points. However, given the limited number of towed receivers, their relatively short offsets from the transmitter, and the diffusive nature of low frequency EM fields, the resistive features are quite smoothly resolved in depth. For both survey regions, the joint inversions of both CSEM and MT data are significantly improved over either of the individual data set inversions, showing the highest lateral and depth resolution and also the strongest resistivity contrasts. Thus, our preferred interpretations are based on the joint inversion models.

We must also consider the possibility that the seafloor conductivity is anisotropic. While MT data are primarily sensitive to horizontal resistivity, the vertical loops of current created by the CSEM transmitter make the towed receiver CSEM data sensitive to both horizontal and vertical resistivity anisotropy. However, all of our independent and joint inversions for isotropic resistivity fit the respective data sets well, so from a data fitting point of view anisotropy is not required. Further, we do not see any anisotropic artifacts such as alternating horizontal bands of resistors and conductors that are characteristic of isotropic inversions of strongly anisotropic data^40^. Therefore, we conclude that isotropic modeling is sufficient for these data.

The distal edge of the resistive zone 90 km offshore Martha’s Vineyard is in close proximity to the seaward limit of the towed CSEM data and so it is worthwhile to examine whether the data require the resistor to end at this location or if the resistor ends because of the truncation of CSEM data. We tested this by running forward models of our preferred inversion model, but with the resistor extending all the way to the shelf edge, representing about 4.5 km^2^ or 9 km^2^ of additional cross sectional area, so that its impact on the MT responses on the outer shelf could be tested (Extended Data Fig. 9). We tested a range of resistivity values (5–100 ohm-m) for these extensions and tabulated the resulting RMS misfit increases for the three MT stations without overlapping CSEM data (M07-M10) in Extended Data Table 1. All resistivity thickness combinations result in an RMS increase greater than our error floor of 10%, indicating it is unlikely fresh to brackish water exists further beyond our interpretation (Fig. 2a).

For our comparison of salinity and bulk resistivity we used a two-stage process. First bulk resistivity was converted into pore fluid resistivity using Archie’s Law, which relates bulk resistivity and porosity to pore fluid resistivity^12^. We use a cementation exponent of *m* = 2 for our Archie’s Law calculation, which is a typical value for seafloor sediments comprised of sands and clays^41^. Porosity was assumed to be 30–60% based on the range encountered in the boreholes off New Jersey^9^. The pore fluid resistivity was then converted into salinity using the Practical Salinity Scale 1978^18^, which is commonly used to convert standard oceanographic seawater conductivity (the inverse of resistivity) measurements into a unitless salinity value. We use a temperature of 10 °C in converting the pore fluid resistivity to salinity, which is reasonable based on temperature measurements from sea-bottom expendable bathy thermographs (XBTs) during data collection. If we use *m* = 1.5 or *m* = 3 for our Archie’s calculations, the salinity values corresponding to our resistive zones slightly decrease and increase, respectively (see Extended Data Table 2), but do not change our overall interpretation of submarine groundwater distributions.

## Supplementary information

Extended Data Figures and Tables

## Data Availability

Upon publication, all data that were analyzed in this study will be made available at https://emlab.ldeo.columbia.edu/index.php/projects/freshwater/.

## Code Availability

The MARE2DEM code used to invert the data is available at http://mare2dem.ucsd.edu.
