# Supplementary material for: Aquifer systems extending far offshore on the U.S. Atlantic margin
Source: Sci Rep. 2019 Jun 18;9:8709. doi: 10.1038/s41598-019-44611-7 (PMC6582133; doi:10.1038/s41598-019-44611-7)
Supplement: Supplementary file 1 — Extended Data Figures and Tables [file 41598_2019_44611_MOESM1_ESM.docx]

**Aquifer systems extending far offshore on the U.S. Atlantic margin**

Chloe Gustafson^1*^, Kerry Key^1^ and Rob L. Evans^2^

^1^ Lamont-Doherty Earth Observatory, Columbia University, Palisades, New York, USA.

^2^ Department of Geology and Geophysics, Woods Hole Oceanographic Institution, Woods Hole, Massachusetts, USA.

*****Corresponding author: cdg2152@columbia.edu

**Extended Data Figures and Tables**

**Extended Data Fig. 1 | Comparison of Martha’s Vineyard independent and joint data inversion models. a-c,** (a) MT data only, (b) surface-towed CSEM data only, and (c) joint CSEM-MT resistivity models for the Martha’s Vineyard shore-to-shelf profile. MT station names are labeled in (a). All inversions fit their respective data to RMS 1.0.

**Extended Data Fig. 2 | Martha’s Vineyard MT data and data fits.** Apparent resistivity and phase data (dots) with corresponding joint-inversion model fits (lines) plotted for each receiver location. Transverse-electric polarization data are shown in blue and transverse magnetic data are shown in red. Sites are ordered from nearest shore to furthest shelf-ward.


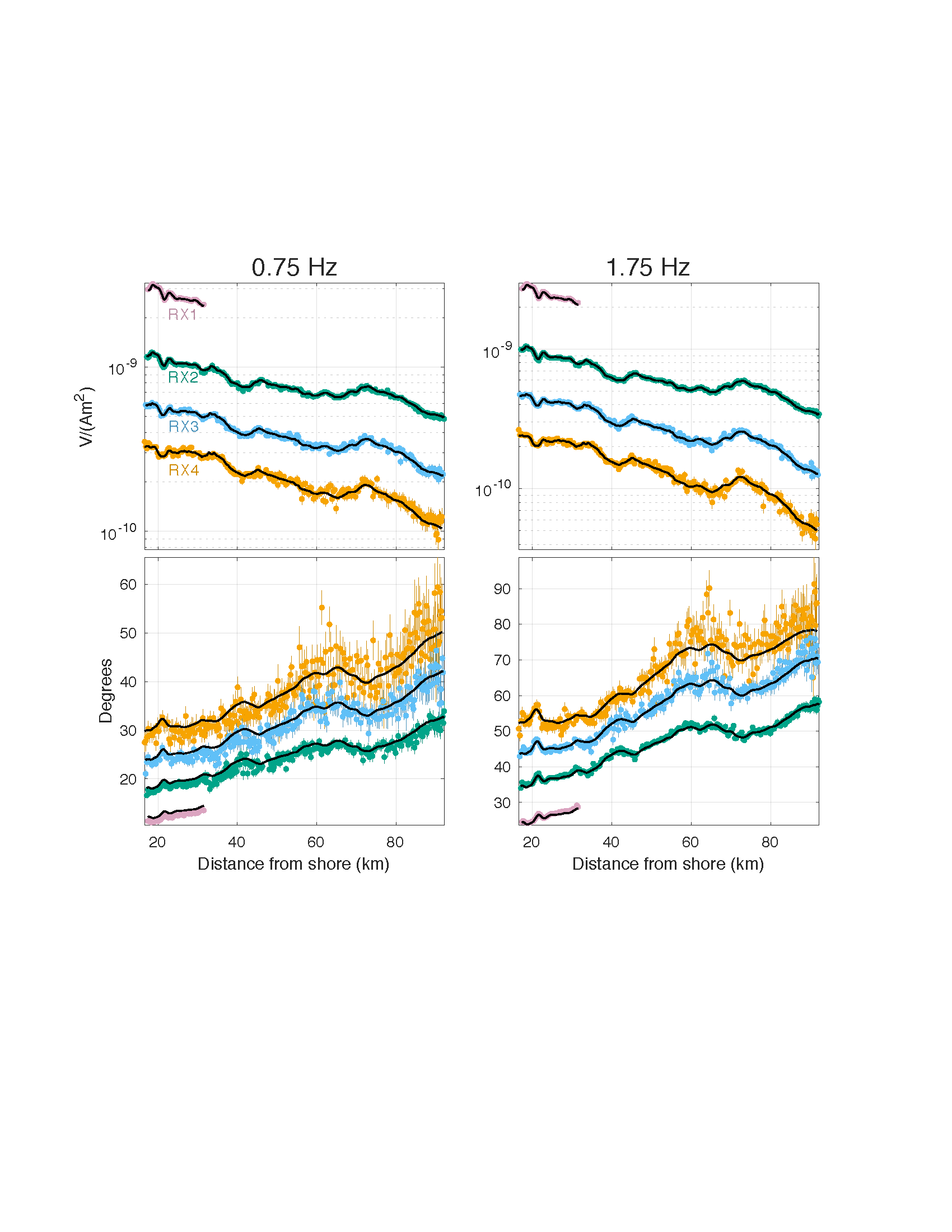


**Extended Data Fig. 3 | Martha’s Vineyard surface-towed CSEM data and data fits.** Amplitude (top row) and phase (bottom row) data (dots) for 0.75 Hz and 1.75 Hz transmissions are shown for receiver offsets of 600m (RX1), 870m (RX2), 1120m (RX3), and 1380m (RX4) with corresponding uncertainty bars (vertical lines). The amplitude data are the measured electric fields (V) normalized by the source dipole moment (Am^2^). Model responses for the joint inversion model (Fig. 2) are shown as black lines and fit the CSEM data to RMS 1.0.

**Extended Data Fig. 4 | Comparison of New Jersey independent and joint data inversion models. a-c,** (a) MT data only, (b) surface-towed CSEM data only, and (c) joint CSEM-MT resistivity models for the New Jersey shore-to-shelf profile. MT station names are labeled in (a). All inversions fit their respective data to RMS 1.0.

**Extended Data Fig. 5 | New Jersey MT data and data fits.** Amplitude and phase response functions (dots) with corresponding 2D model fits (lines) plotted for each receiver location. Transverse-electric polarization data are shown in blue and transverse magnetic data are shown in red. Sites are ordered from nearest shore to furthest shelf-ward.


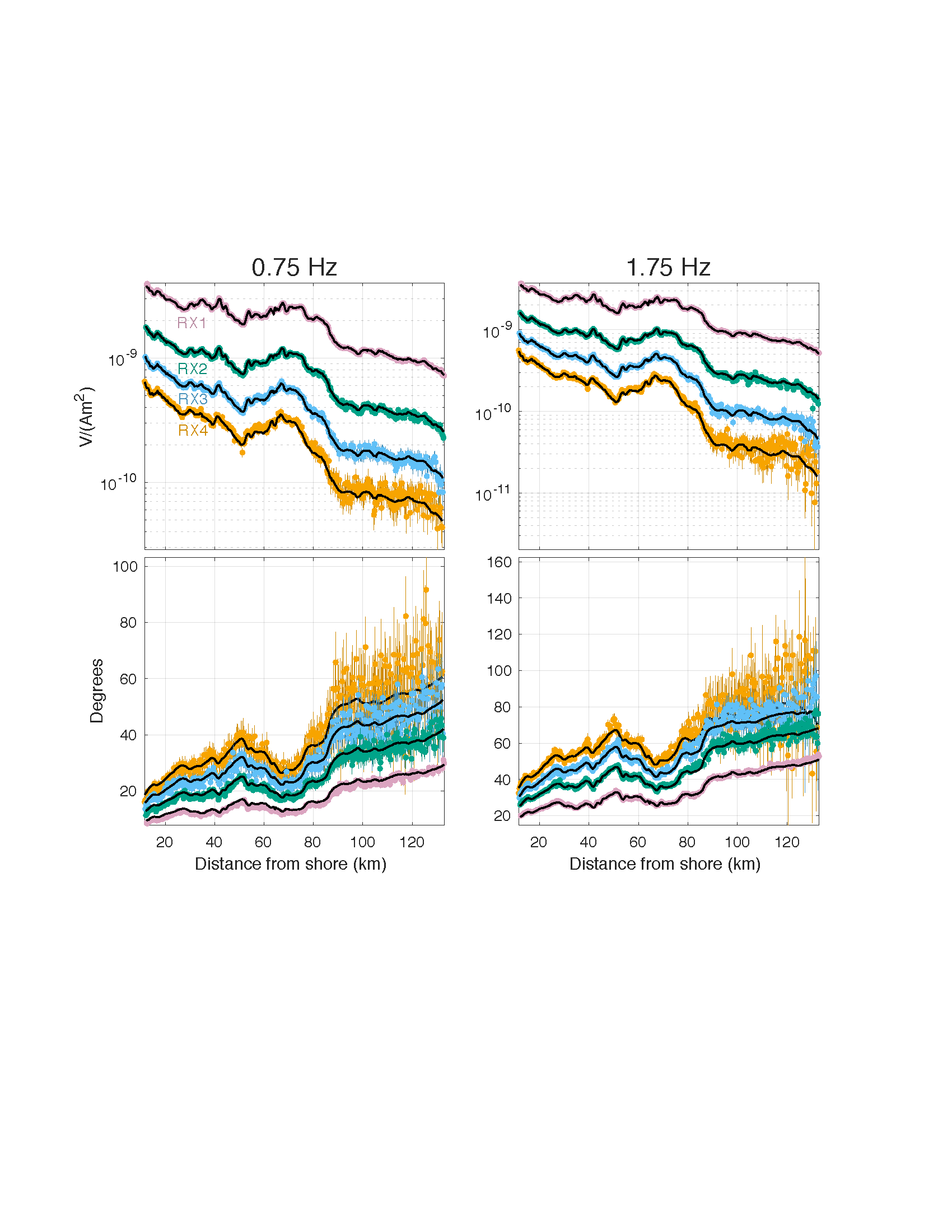


**Extended Data Fig. 6 | New Jersey surface-towed CSEM data and data fits.** Amplitude (top row) and phase (bottom row) data (dots) for 0.75 Hz and 1.75 Hz transmissions are shown for receiver offsets of 600m (RX1), 870m (RX2), 1120m (RX3), and 1380m (RX4) with corresponding uncertainty bars (vertical lines). The amplitude data are the measured electric fields (V) normalized by the source dipole moment (Am^2^). Model responses for the joint inversion model (Fig. 2) are shown as black lines and fit the CSEM data to RMS 1.0.

**Extended Data Fig. 7 | CSEM data noise floor.** Amplitude (dots) for (a) 0.75 Hz and (b) 1.75 Hz transmissions for receiver offsets of 600 m (RX1), 870 m (RX2), 1120 m (RX3), and 1380 m (RX4) with corresponding uncertainty bars (vertical lines) plotted as a function of time. The transmitter was off prior to 13:00:00 UTC. We plot the noise floor (solid black line) as the average of the amplitudes during times when the transmitter was turned off. The measured signal appears as random noise with large uncertainty bars when the transmitter is turned off. When the transmitter is turned on the measured signal is coherent with small uncertainty bars and over an order of magnitude higher than the noise floor for all receivers at both frequency transmissions.

­

**Extended Data Fig. 8 | All New Jersey 2-D resistivity models.** All survey profiles shown in perspective as a fence diagram. The color scale is the same as previous models, displaying the log resistivity. The vertical axis for all models is kilometers below sea level, with all models displaying 0-0.8 km below sea level. Lines crossing the resistive zone indicate the aquifer extends shore-parallel in both directions and shows good agreement with the presence of a deeper conductive zone from about 70 km and further offshore. Crossing lines near the shelf edge (right side) confirm the resistive zone does not extend further shelf-ward.

**Extended Data Fig. 9 | Martha’s Vineyard extension outline.** Black dashed box represents the area where resistivity values were modified in order to study the induced changes in the MT response functions and corresponding data fits.

| **Resistivity (ohm-m)** | **RMS increase**  **Extension a** | **RMS increase**  **Extension b** |
| --- | --- | --- |
| 5 | 12.9% | 53.49% |
| 10 | 20.5% | 78.2% |
| 25 | 26.2% | 95.3% |
| 50 | 27.3% | 100.8% |
| 100 | 27.5% | 103.8% |

**Extended Data Table 1 | Martha’s Vineyard resistivity extent test**. Resistivity values assigned to the Martha’s Vineyard resistor extension and corresponding percent changes in RMS misfit value for the MT responses. This test suggests it is unlikely our interpreted zone of low-salinity water extends further toward the shelf edge.

| **Cementation exponent m** | **Min salinity for 30% porosity** | **Max salinity for 30% porosity** | **Min salinity for 60% porosity** | **Max salinity for 60% porosity** |
| --- | --- | --- | --- | --- |
| 1.5 | 0.38 | 170 | 0.13 | 48 |
| 2 | 0.71 | 416 | 0.17 | 64 |
| 3 | 2.5 | 5098 | 0.29 | 120.7 |

**Extended Data Table 2 | Inferred salinity values given different cementation exponents in Archie’s Law calculation**. Minimum and maximum salinity values for porosities 30% and 60% given cementation exponents *m* = 1.5, *m* = 2 and *m* = 3. Substituting to *m* = 1.5 and *m* = 3 results in slightly lower and higher predicted minimum salinities, respectively, however, still supports our conclusion that an extensive low-salinity aquifer underlies the eastern U.S. Atlantic continental shelf.
